# Immune Checkpoint Inhibitors and the Heart

**DOI:** 10.3389/fcvm.2021.726426

**Published:** 2021-09-29

**Authors:** Diana Larisa Mocan-Hognogi, Sebastian Trancǎ, Anca Daniela Farcaş, Radu Florin Mocan-Hognogi, Andrada Viorica Pârvu, Anca Simona Bojan

**Affiliations:** ^1^Internal Medicine Department, “Iuliu Haţieganu” University of Medicine and Pharmacy, Cluj-Napoca, Romania; ^2^1st Cardiology Department, Emergency Clinical County Hospital, Cluj-Napoca, Romania; ^3^Surgery Department, “Iuliu Haţieganu” University of Medicine and Pharmacy, Cluj-Napoca, Romania; ^4^Intensive Care Department, Emergency Clinical County Hospital, Cluj-Napoca, Romania; ^5^Mother and Child Department, “Iuliu Haţieganu” University of Medicine and Pharmacy, Cluj-Napoca, Romania; ^6^2nd Gynecology Department, Emergency Clinical County Hospital, Cluj-Napoca, Romania; ^7^Oncology Department, “Iuliu Haţieganu” University of Medicine and Pharmacy, Cluj-Napoca, Romania; ^8^Hematological Department, “Prof. Dr. Ioan Chiricuţǎ” Oncology Institute, Cluj-Napoca, Romania

**Keywords:** immune checkpoint inhibitors, chemotherapy, cardiotoxicity, immune-related adverse events, cancer, CTLA-4, PD-L1

## Abstract

Immune checkpoint inhibitors (ICIs) represent a break-through treatment for a large number of cancer types. This treatment is increasingly being recommended. ICIs are prescribed for primary tumours and for metastases, adjuvant/neo-adjuvant therapy. Thus, there is an increased need for expertise in the field, including the ways of response and toxicities related to them. ICIs become toxic because of the removal of self-tolerance, which in turn induces autoimmune processes that affect every organ. However, when relating to the heart, it has been noticed to be leading to acute heart failure and even death caused by various mechanisms, such as: myocarditis, pericarditis, arrhythmia, and Takotsubo cardiomyopathy. This review aims to address the above issues by focusing on the latest findings on the topic, by adding some insights on the mechanism of action of ICIs with a special focus on the myocardial tissue, by providing information on clinical manifestations, diagnosis and (wherever possible) treatment of the cardiotoxic events related to this therapy. The information is expanding and in many cases, the articles we found refer mainly to case-presentations and studies conducted on small populations. However, we consider that it is worthwhile to raise awareness of this new treatment, especially since it is widely now and it provides a significant increase in the survival rate in patients who receive it.

## Introduction

The immune system plays a paramount role in maintaining the balance between self and non-self cells, but it might have a serious problem when having to make a distinction between malignant and benign cells. To be able to do this, it needs to have the ability to eliminate the tumour cells, which in turn always try to evade the immune system and proliferate. These mechanisms are known as “immune editing” (1). As we can easily conclude, cancer develops secondary to the toleration of the malignant cells because tumour cells are able to cause an overexpression of the checkpoint proteins that protect them from being destroyed by the immune system. Thus, in order to be able to maintain the balance, this system needs both inhibitory and stimulating signals. First of all, it needs a stimulator in order for the system to start producing immune factors and then it needs inhibitors so that the system does not start overreacting and hence self-tissue destroying (1).

Over the last years a large variety of cancer types were targeted through checkpoint inhibition: melanoma, lung, head and neck, renal cell, urothelial, Hodgkin's lymphoma, etc. However, the problem with this type of immunological treatment is the adverse reactions that can occur on different levels: brain, skin, gastrointestinal system, liver, pancreas, lungs, kidneys, endocrine system, neurologic system, haematologic system, ophtalmologic level, cardiac system and musculoskeletal level as well (1). These effects range from minor to major.

Recently, several authors have reported cases of severe cardiotoxicity in patients treated with immunotherapy, but their incidence is still low maybe because, until now there have not been conducted large populational studies on these effects. Given that cases of severe heart failure and death are reported, cardiologists and oncologists give special consideration to this therapy.

## Types of Checkpoint Inhibitors

One of the pivotal modulators and effectors of the immune system are T cell lymphocytes. Antigen presenting cells (APCs) activate naïve T cells through the interaction between MHC (major histocompatibility complex) expressed on the APCs and the T cell receptor (TCR). Furthermore, there are several other stimulatory signals, namely: CD28, CD80 (B7-1) or CD86 (B7-2), which are also essential for the activation of T cell lymphocytes. But to prevent the hyperactivation of the immune system, they need to be regulated by immune checkpoints (2).

Several major classes of ICIs have been used up until now, namely:

- monoclonal antibodies against PD-1–programmed cell death protein-1 (pembrolizumab, nivolumab, cemiplimab, dostralimab) and its ligand PD-L1 (atezolizumab, avelumab, durvalumab)
- monoclonal antibodies against cytotoxic T lymphocyte-associated antigen 4 (CTLA-4): ipilimumab, tremelimumab, quavonlimab
- combination of CTLA-4 and PD-1: ipilimumab and cemiplimab; Ipilimumab + pembrolizumab, Tremelimumab + durvalumab.
- novel checkpoint inhibitors targeting: lymphocyte activation gene-3 (LAG-3), T cell immunoglobulin and mucin-domain containing-3 (TIM-3), B and T cell lymphocyte attenuator (BTLA), T cell immunoglobulin and ITIM domain (TGIT), V domain Ig suppressor of T cell activation (VISTA) and B7 homologue 3 protein (B7-H3) (3).

## Mechanism of Action

The main goal of the checkpoint inhibitors is to decrease autoimmunity by activating more non-T cells as opposed to T regulatory cells, thus targeting tumour cells (2). There are many types of tumour, that can benefit from treatment with ICI, as shown in Table 1.

**Table 1:** Types of checkpoint inhibitors and targeted cancers.

**Class of ICI**	**Drug**	**Types of targeted cancers**
CTLA-4-i	Ipilimumab	Melanoma
PD1-i	Nivolumab	Melanoma, NSCLC, SLCL, RCC, HCC, Hodgkin's lymphoma, head and neck cancer, metastatic colorectal cancer, urothelial carcinoma
	Pembrolizumab	Melanoma, NSCLC, Hodgkin's lymphoma, urothelial carcinoma, gastric cancer, large B cell lymphoma primarily mediastinal location, cervical cancer
	Cemiplimab	Metastatic cutaneous squamous cell carcinoma
PD-L1-i	Atezolizumab	NSCL, urothelial carcinoma
	Avelumab	Meckel cell carcinoma, urothelial carcinoma
	Durvalumab	Urothelial carcinoma, NSCLC
Combination of PD1-i and CTLA-4 i	Ipilimumab+ Nivolumab	Colorectal cancer (some subtypes), melanoma and RCC

*PD-1-I, Programmed cell death ligand 1 inhibitor; CTLA-4 I, cytotoxic T lymphocyte antigen 4 inhibitor; HCC, hepatocellular carcinoma; ICI, immune checkpoint inhibitor; NSCLC, non-small cell lung cancer; RCC, renal cell carcinoma; SCLC, small cell lung cancer [adapted after Zhou et al. (4) and Tajiri et al. (5)]*.

Several events allow the immune system to target tumour cells, as follows (6):

1. the priming phase consists in the amplification of the T cell response. This cycle begins when the dendritic cells recognise cancer cell antigens via a major histocompatibility complex, thus priming the activation of effector T cells onto cancer cells.
2. The effector phase: activated effector T cells travel and infiltrate the tumour starting destruction of cancer cells. This activity is made possible through the interaction between the T cell receptor (TCR) and cognate antigen bound to MHC. Subsequently, more cancer cell antigens are released and a mechanism of positive feedback expands the immunity of T cells to tumour cells.

The main goal of checkpoint inhibitors is to decrease autoimmunity/autoimmune activity by activating more non-T cells as opposed to T regulatory cells, thus targeting tumour cells (2) Numerous types of tumour can benefit from treatment with ICI, as shown in Table 1.

Some of the mechanisms of adaptive immune resistance include:

- down-regulation of major histocompatibility complex antigen expression,
- secretion of immunosuppressive cytokines,
- negative regulation of cytotoxic CD8+ T cells through checkpoint inhibition (7).

### PD-L1

PD-L1 is expressed on the B lymphocyte membrane and other antigen presenting cells (APCs) such as macrophages and dendritic cells. PD-L1 is the programmed cell death ligand expressed in tumour cells. PD-1 action revolves around the tumour environment and it prevents T cells from expressing their function (8). They act mostly in the effector phase, and the blockade occurs mainly at the tissue level and in the microenvironment of the tumour (9).

The PD-1/PD-L1 duo reduces the cytokine production and the T lymphocyte proliferation and survival. These actions help blocking the negative regulatory signalling pathway, thus enhancing the actions of the immune system against tumours. They do this by activating earlier primed T cells, which have lost previous effector and proliferative functions (4, 10, 11). After activation, T cells, B cells, natural killer cells, natural killer T cells, macrophages and dendritic cells express PD-1 on their surface (2). Several types of cells express PD-L1, namely: the haematopoietic and non-haematopoietic cells such as hepatocytes, astrocytes, epithelial cells, muscle cells (including cardiomyocytes), vascular endothelial cells and pancreatic cells (2). Many authors have also concluded that the tumour expression of PD-L1 is associated with a poor prognosis.

### CTLA-4

CTLA-4 is found in the intracellular vesicles only on activated T cells and is responsible for the amplitude of T cell activation (4). It belongs to the B7/CD28 family and acts by indirectly lowering signalling through the co-stimulatory receptor CD28, which also restores T cell-three-signal activation in the tumour, draining lymph nodes (9). It is translocated to the cell surface in response to T-cell receptor (TCR) activation. CD28 and/or IL2 co-stimulate their upregulation. It competes with CD28 for binding with B7 ligands (CD80, CD86), for which it also has higher affinity (10, 11). This leads to the suppression of the priming phase. CTLA-4 also suppresses regulatory T cells (9).

Naturally, cancer cells start expressing PD-1/PD-L2 as they try to protect themselves and survive. It is understandable that targeting PD-1, PD-L1, PD-L2, CTLA-4 leads to an enhanced immunological response against tumour cells.

## Risk Factors for Cardiac Adverse Events Associated With ICIs

Authors have not concluded yet on the risk factors that predispose to important cardiac toxicity, in patients treated with ICIs. However, some specialists have pointed out some elements of predisposition (Table 2) but they have not been confirmed yet on large cohorts. Table 2 shows a list of possible risk factors identified more frequently in patients who have developed immune-related adverse events (IRAEs). Therefore, we believe that cardio-oncology specialists should give special attention and perform frequent follow-up examination during treatment with ICIs.

**Table 2:** Risk factors for developing cardiac IRAEs [adapted after Varricchi et al. (2) and Zhou et al. (4)].

Therapy with combination of ICIs
Detection of skeletal myositis (usually precedes myocarditis)
Lung cancer (combination of radiotherapy and ICIs)
Autoimmune disorders (rheumatoid arthritis, systemic lupus erythematosus, sarcoidosis)
Male gender
Concomitant use of anthracyclines, anti-ErbB2 drugs, Raf and MEK inhibitors, VEGF tyrosine kinase inhibitors
Genetic polymorphism of CTLA-4, PD-1, PD-L1; activation of T-cell clones against cardiac antigens
Cardiotoxic therapies
Decreased global longitudinal strain - GLS (hypertension, coronary artery disease, heart failure, myocardial infarction, myocarditis, diabetes mellitus, dyslipidemia)
ECG conduction disease
Flu vaccination – protection from IRAEs

## Adverse Events Associated With the Treatment With Immune Checkpoint Inhibitors

The current literature has shown that the treatment with ICIs, used as standard therapy for cancer patients, is often accompanied by multiple immune-related adverse events (IRAEs) such as colitis, thyroid hormones imbalance, dermatological, musculoskeletal, gastrointestinal and cardiovascular events. These seem to be correlated with the number of drugs prescribed and used (single class or combination) and occur more frequently during the first 3 months of treatment. They are usually induced by erratically autoreactive T cell activation (12–14). Most of the IRAEs can be antagonised with anti-inflammatory agents such as glucocorticoids and in some cases more potent therapies such as infliximab (an anti-TNF alpha receptor agent) or mycophenolate (an inhibitor of purine synthesis in T and B-cells) (15). However, some IRAEs do not respond to any of these treatments.

The exact mechanisms of cardiac involvement still require clarification. Some cardiac pathologies might just be coincidental with the malignancy in a patient, and therefore it is rather difficult to identify cardiac adverse events associated with the ICIs therapy but this is of paramount importance however, as such a condition can be profoundly serious and even life-threatening, having the potential to lead to death.

Elosta et al. demonstrated in a meta-analysis, which included 28 clinical trials, that IRAEs occur more frequently in patients treated with CTLA-4 inhibitors as compared to PD-1 and PD-L1 blockers (53.8, 26.5, and 17.1%, respectively) (1). Consequently, they have concluded that targeting immune and regulatory T cells is accompanied by a higher incidence of adverse events.

## Mechanism of Immune Cardiotoxicity

A 2018 paper by Xiaoxiao et al. showed that during a period of 10 years the total number of cardiac IRAEs declared in the WHO global database counted 31,321 (16). The autopsy and histological specimens from patients or animal models treated with ICIs have shown that myocarditis is a major cardiac lesion.

Other types of cardiovascular adverse events also exist, namely: pericardial effusion, arrhythmias (out of which supraventricular tachycardia is more commonly encountered), acute coronary syndrome, vasculitis (e.g., temporal arteritis or rheumatic polymyalgia) (17).

In healthy individuals, the thymus regulates the number of autoreactive T cells that are released in the periphery. According to this “central tolerance” some of them are deleted and others are distributed in the periphery according to “peripheral tolerance.” The “immunotolerance” results from the downregulation of T cell activation by means of the competition between CTLA-4 and CD28 (2). Once this tolerance is removed however, the immune system develops a state of hyperactivity with subsequent macrophage-mediated toxicity and production of antibodies from activated B cells alongside a low level of T reactive cells (18).

Moreover, the interval of time required for toxicity to occur has not been exactly established yet; besides, it seems not to follow any pattern driven either by type or by target. In addition, mechanisms differ even in patients treated with the same agent.

### Types of Immune Checkpoint Inhibitors-Related Cardiac Events

**Figure 1:**
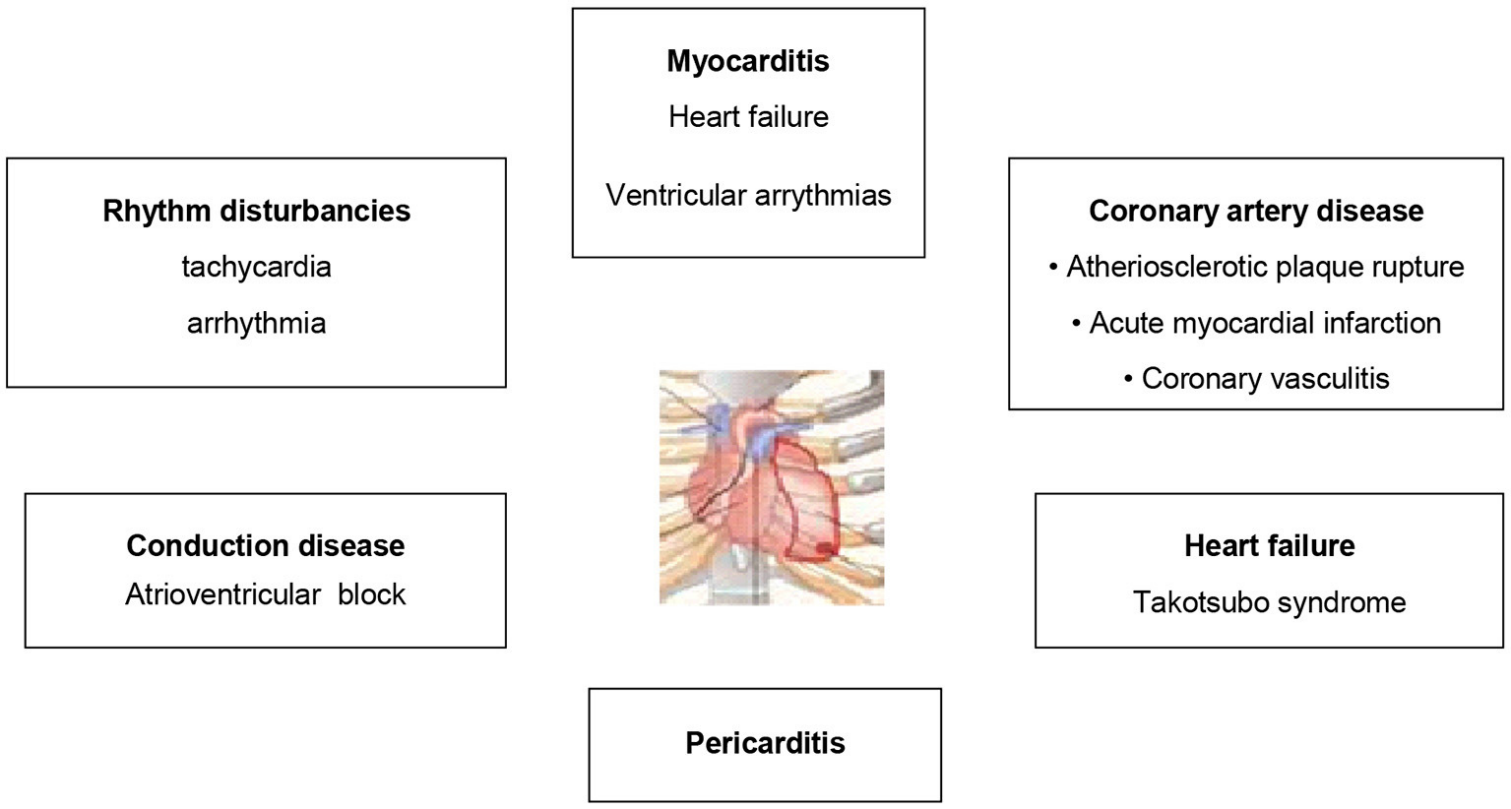
Main clinical types of cardiac involvement during treatment with immune checkpoint inhibitors.

Main clinical cardiotoxic events are shown in Figure 1.

#### Myocarditis

The predominant histopathological trait in myocarditis is lymphocytic infiltrates in the myocardium and the conduction system. They are mostly represented by CD3, CD4+/CD8+ lymphocytes and by some CD68 cells (macrophages) and multinuclear giant cellular infiltration (16, 19–21). This finding was also demonstrated in murine models. The development of severe myocarditis was observed in CTLA-4 -deficient mice. They also proved to have massive T cell infiltration (22). Compared to them, another type of behaviour was found in PD-1 -deficient mice. Thus, those with BALB/c background developed autoimmune dilated cardiomyopathy (23), whereas PD-1 -deficient autoimmune-prone MRL mice showed an important CD4+ and CD8+ T cell infiltration (24). Similar findings of severe myocarditis were reported in PD-L1 -deficient MRL mice (22). All things considered, the severity of the clinical manifestation of this autoimmune disease relates to the disruption in the PD-1/PD-L1 pathway and may be attributed to polymorphism in specific genes as highlighted on the murine model of the PD-L1/MRL mice. Authors state that similar assumptions can also be made in human subjects (22).

Myocarditis was observed at a median of 27 days (range 5–155) from the initial dose of ICI therapy but apparently most cases emerge during the first 6 weeks (25). Unfortunately, there is limited information about the exact onset time of the disease as the number of cases included in the studies so far is limited, so the data is uncertain.

The severity of the myocardial disease was positively correlated with the number of doses of anti-CTLA-1 but not with that of anti PD-1/antiPD-L1 antibodies (26, 27). However, there were also reports of cases, in which patients developed this condition after they were administered only one dose of anti-CTLA-1 antibodies.

**Figure 2:**
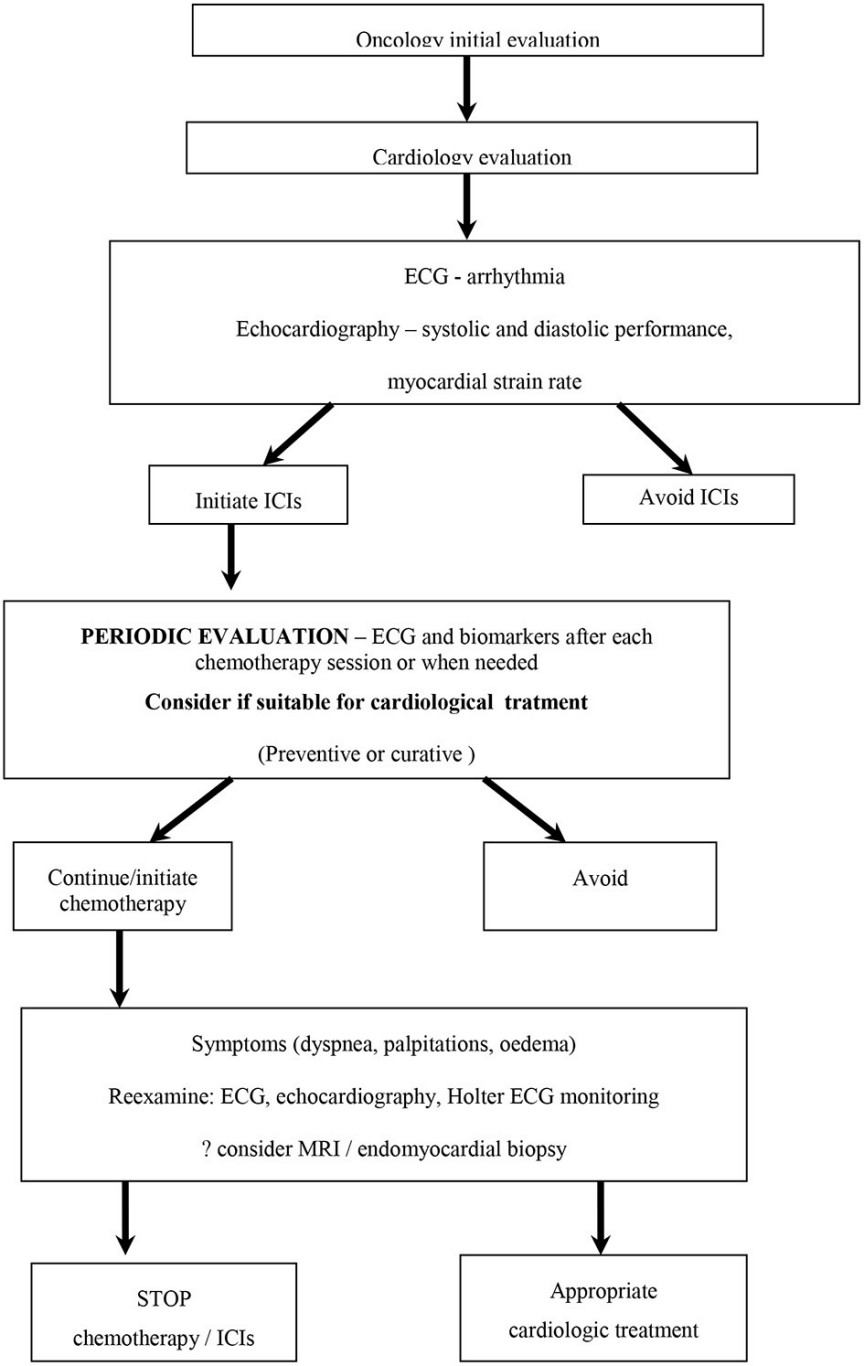
Algorithm for evaluating the patient who will be treated with immune checkpoint inhibitors [adapted after Liu et al. (28)].

Some patients may be asymptomatic but some develop signs and symptoms of heart failure. The conduction system may also be involved and therefore patients can present with conduction abnormalities, such as block of different types and degrees. Moreover, malignant arrhythmias can occur (2). Hence, sudden cardiac death is also possible. Unfortunately, no algorithms have been found so far that allow identification of patients at risk. Because of the heterogeneity of the clinical picture and time of onset, it is extremely important to develop tools for the early detection of ICI-related myocarditis so that patients can receive the proper treatment. Therefore, the introduction of biomarkers related to myocyte damage would be a promising step forward. Some authors suggest the measuring of troponin levels, while others consider NT-proBNP to be helpful (28). One should however, also carefully assess whether the elevation of these biomarkers cloud also be caused by other concomitant cardiac conditions. Therefore, perhaps a dynamic assessment, which includes a series of periodical clinical evaluation combined with an EKG, biomarkers and echocardiography, might be helpful to allow the patient to be properly referred to the cardio-oncology team for assessing whether further investigations and/or treatment are required (MRI, PET-scan) [Figure 2; (29)].

Chen et al. reported that the degree of troponin elevation could predict cardiovascular death, cardiogenic shock and cardiac arrest while persistent troponin elevation was a significant predictor of a 4-fold increased risk for major adverse cardiaovascular events (MACE) (30).

ECG abnormalities in cancer patients treated with ICIs include sinus tachycardia, ventricular and supraventricular arrhythmias, bundle branch block, complete AV block and ventricular tachycardia, therefore basic ECG is also important to be performed baseline and during treatment. Unfortunately, all of these are non-specific and the ECG examination is often times normal in myocarditis (19, 31).

The study of Mahmood et al. on patients with ICI-related myocarditis found abnormal ECG in 89% of the patients, NT-proBNP elevation in 66% of them, while the left ventricular ejection fraction (LVEF) only in 49% and concluded that LVEF is not a suitable diagnostic item for these patients (32). Similar results where reported by Awadalla et al., who showed that 60% of the patients presenting with myocarditis following ICIs had preserved EF in spite of a large amount of affected myocardium (33).

In contrast, the study published by Escudier et al. found LV systolic dysfunction was found in 79% of the patients (34). These conflicting results suggest that LVEF alone might not be a reliable tool to assess myocarditis (30). Thus, in order to identify the myocardial involvement and to establish risk criteria, the global longitudinal strain (GLS) was proposed for monitoring cancer patients who receive chemotherapy (35, 36) because it was shown that GLS could identify myocardial involvement even in the context of preserved EF. In patients with myocarditis after ICIs, Awadalla et al. have reported that GLS is reduced in all myocarditis patients regardless of decreased or preserved EF at baseline. GLS decreases during hospitalisation and also proved to have predictive power because every decrease in GLS was associated with an increase in MACE (1.5-fold in patients with decreased EF and 4-fold in patients with preserved EF) (33).

Mincu et al. showed that monitoring GLS in melanoma patients could identify ICIs- induced subclinical left ventricular dysfunction (in the absence of myocarditis) and extracardiac adverse events during the first month of treatment, whereas ejection fraction monitoring could only identify the radial strain but not the circumferential strain (37).

Kasner et al. showed that patients with chronic myocarditis have reduced GLS even with preserved EF (38).

Further, GLS was found to have superior diagnostic performance (sensitivity, specificity, and accuracy of 82, 70, and 76%, respectively) when compared to cMRI based on the Lake Louise criteria (sensitivity, specificity, and accuracy of 54, 71, and 67%, respectively), while their combination further increased the diagnostic performance (sensitivity, specificity, and accuracy of 96, 55, and 75%, respectively) (38).

Cardiac magnetic resonance is the non-invasive technique commonly used in myocarditis, being also helpful (76% sensitivity and 96% specificity) (39). The features of ICIs induced myocarditis are slightly different from those usually found in other types of myocarditis. In some cases, no inflammation, no fibrosis or scarring can be found (32, 40–42).

Escoudier et al. reported myocardial ischemia in 33% of the patients and late gadolinium enhancement (LGE) in 23%, but the number of subjects included in the study was only 15 (39). Mahmood et al. studied 35 cases and found LGE in 74% of them. The discrepancies are high and consequently there is a need to evaluate them on larger cohort studies (34).

The gold-standard in the diagnosis of myocarditis remains the endomyocardial biopsy because it can provide evidence of the lymphocytic infiltrate, CD4 and CD^*^ T cells, CD68 macrophages, rare CD20 cells and plasmocytes with no evidence of eosinophilic granulomas or giant cells (40, 41, 43, 44) because the mechanism is a direct injury by hiperactivated T cells (30).

Recently, Finke et al. have used FAPI PET/CT in patients treated with ICIs and showed that it can be useful for the early detection of myocarditis and cardiac risk stratification (in combination with biomarkers, ECG and echocardiography (45).

With regard to management, the treatment usually consists of corticosteroids and immunotherapy (immunosuppressive agents, high-dose intravenous immunoglobulin, immunoabsorption, and plasmapheresis) for non-responders to steroids (22, 46).

Immunotherapies for cancer are relatively new, and therefore long-term data regarding prognosis in patients with cardiotoxicity following ICIs treatment is scarce. However, this issue has been addressed in some studies that have found and reported high fatality rates. For example, a systematic review that included 99 patients has found a fatality rate of 35%. Other observational studies have concluded that there is a 50% risk of major cardiac adverse events in ICIs related myocarditis in comparison to non-ICIs related myocarditis (47–49).

#### Pericarditis

Pericarditis is another possible complication of ICIs therapy; it can occur even after the first dose but usually it occurs 6–11 months after the initial dose of the ICI treatment. Patients can develop either tamponade, or effusive-constrictive pericarditis. The exact mechanism that leads to the pericardial effusion has not been fully explained yet; it might be inflammation. In a systematic review paper that included 705 cases of ICI-associated pericardial disease, the authors have stated that this condition is not as rare as initially believed but they have mentioned that there might be some biases coming from the fact that some malignancies complicate with pericardial effusion even in the absence of immunotherapy. Hence, the diagnosis of ICIs-related pericardial effusion is challenging (50).

ICIs- associated pericardial disease mainly affects men (60%) it was more frequently associated with anti-PD-1/PD-L1 regimens and combination therapy did not increase this. Moreover, the various types of cancer and the different ICIs approved for these specific tumours might influence the occurrence of the pericardial disease. Another confounding factor that might alter the percentage of pericarditis is the use of radiation in conjunction with immunotherapy. It appears that radiation primes an endogenous antigen specific immune response (17, 50). They expose potential shared antigens to T cell recognition, and this in turn might contribute to the development of pericarditis (30). Some studies have attributed this adverse event to nivolumab therapy for lung cancer. Some patients with previous tuberculosis have experienced a reactivation of this condition apparently because of host induced hypersensitivity response (51–53).

Clinically, these patients present similar symptoms to those described in pericarditis of other causes: chest pain, shortness of breath, etc., though in some cases, it might rapidly develop into respiratory failure. The ECG shows low QRS voltage, PR segment depression, and inversion of T waves. The echocardiography is a useful tool to detect the pericardial effusion, but in some cases CT and MRI were used. Troponin was usually elevated when pericarditis was accompanied by myocarditis (30, 34, 52–55).

We have found several articles, consisting of case-reports and studies conducted on small cohorts. In all cases, pericardiocentesis was the treatment of choice; the pericardial fluid analysis showed leukocytes with lymphocyte predominance and no signs of malignant cells (51–55).

#### Arrhythmias

As hypothesised before, MRI tests conducted in patients with myocarditis showed signs of inflammation. This in turn contributes to a significant heterogeneity in the myocardium, which can lead to a multitude of rhythm and conduction disturbances. Escoudier et al. reported atrial fibrillation in 30% of patients, ventricular arrhythmias in 27% and conduction disturbances in 17% of the patients in their study (34). Some authors mention that the presence of atrial fibrillation should be regarded with caution as it might be due to the ICI treatment itself. However, arrhythmias are more likely to coexist with other conditions such as myocarditis rather than be caused by the ICI treatment itself. We also need to mention that ventricular tachycardia and ventricular fibrillation cause sudden death, and therefore extra care should be given to any of the above signs (56, 57).

Inflammatory T cells infiltrate the conduction system so the ICI- mediated conduction disease is very serious and can be fatal. Puzanov et al. in their article, suggested that all patients receiving ICIs should be regularly screened at baseline and every 1–2 weeks for 6 weeks using an ECG. These patients should be taken into consideration for early pacing, even more so if they also have myocarditis because the progression towards complete AV block is frequent, and there is increased risk for sudden death (11). We conclude that whenever bradycardia or heart block is found, the patient should be referred for Holter ECG monitoring, echo and even an MRI so that the physician can obtain more information about subclinical inflammation or myocarditis allowing an oncology-cardiology team to make the appropriate decision.

It appears that the inflammation secondary to T lymphocytes patchy infiltration in the sinoatrial and atrioventricular nodes is also responsible for atrial fibrillation. In conclusion, the development of atrial fibrillation is directly connected to the treatment with ICIs (49, 58).

An evaluation report made public by the European Medicine Agency revealed the fact that the authors reported 1.3% incidence of tachycardia, 0.4% incidence of arrhythmia and 0.2% incidence of atrial fibrillation in the patients treated with nivolumab in combination with ipilimumab (59).

#### Takotsubo Syndrome

Also known as “the broken heart syndrome,” this condition consists of left ventricular dysfunction accompanied by wall motion abnormalities, which usually involves the apical and mid-myocardium portion of the left ventricle. This is transient and it occurs in the absence of a significant atherosclerotic disease. The mechanism underlying this condition is unknown but there are several suppositions: one is the direct action of ICIs on coronary arteries, which leads to coronary vasospasm in multiple areas (probably affecting not only large epicardial arteries but also the microvasculature).

Other authors have proposed an interesting mechanism mainly concentrated around a myocardial response to an increased release of catecholamines from the adrenal gland and postganglionic sympathetic nerves (28, 60). The exact mechanism is unclear yet.

Some studies have mentioned a high prevalence (up to 28.5%) of cancer in TTS patients and this subgroup has also been reported to have high mortality rates (61, 62). Data shows that in most of these cases the contractility of the left ventricle is especially poor at the level of the apex, which is also ectatic. This feature is similarly found in non-cancer patients with TTS so it might is not necessarily be related to ICIs. A significant number of patients have been reported to develop the “inverted TTS,” which is basal and mid segment akinesia and minimal/moderate LV systolic dysfunction (63, 64). Apparently, these patients develop TTS later in the course of immunotherapy (15 weeks−8 months) but the alterations are reversible with conventional treatment such as beta-blockers, ACEIs, corticotherapy in conjunction with heart failure treatment (30).

#### Myocardial Infarction

Ischaemic heart disease is a condition accompanied by chronic inflammation. This substantially accelerates plaque rupture, which is the fundamental event that leads to myocardial infarction and stroke. When using ICIs, there are at least 2 mechanisms that have been postulated as being involved in the acute myocardial infarction:

1. The activation of inflammation in preexisting plaques which triggers fibrous cap rupture and therefore acute coronary thrombosis
2. The direct activation of T cell-mediated coronary vasculitis in the absence of atherosclerosis.

The latter mechanism still needs to confirmation. The exact sequence of events is difficult to fully establish as patients with cancer are usually older and with concomitant/ associated cardiovascular disease. Numerous questions still require answers, namely: whether immunotherapy could increase long-term cardiovascular inflammation; whether immunotherapy transiently increases plaque inflammatory activity, which in turn would trigger future acute coronary events. Another question also needs an answer on how acute inflammatory reactions to tumours trigger other events such as activation of platelets and coagulation cascade, which in turn contribute to cardiovascular toxic events (28).

## Management

The management of immunotherapy-related complications requires multiple approaches and depends on the severity of the cardiotoxicity. In a position paper, the Society for Immunotherapy of Cancer (SITC) Toxicity Management Working Group, highlitghts 4 degrees of severity:

1. Abnormal cardiac biomarker testing, including abnormal ECG: it does not require discontinuation or immunotherapy.
2. Abnormal screening tests with mild symptoms: requires management of additional cardiac disease and risk factors.
3. Moderately abnormal testing or symptoms with mild activity: withdrawal of the ICIs therapy; initiation of high-dose prednisolone (1–2 mg/kg).
4. Moderate to severe decompensation that requires intravenous medication or intervention or life-threatening conditions: consider high-dose corticosteroid therapy. Also consider immunoglobulins, infliximab, or anti-thymocyte globulin as second-line therapy (11).

### Discontinuation of the Treatment With Immune Checkpoint Inhibitors

The decision of discontinuation of the ICIs treatment requires a multidisciplinary cardio-oncology approach. We should keep in mind that ICIs have long half-life and cessation of the treatment at one point would not correct the adverse effects at once. This decision also requires certainty that the clinical cardiac complication is related to the treatment with ICIs. In mild ICIs cardiotoxic events, authors conclude that restarting treatment is reasonable after the resolution of cardiotoxicity, but with close surveillance regarding the recurrence of such events. Nevertheless, these decisions are difficult to make and close monitoring by the/a cardio-oncology specialist is mandatory (28).

### Consider Conventional Therapies for Cardiac Events

Whenever necessary, specialists must use other conventional cardiac treatments to manage complications like overt pulmonary oedema (use of diuretics, nitrates), complete AV blocks (use of temporary/permanent pacemakers), ventricular tachyarrhythmias (use of beta-blockers, amiodarone or electrical cardioversion/defibrillation). In extreme cases of cardiogenic shock, the use of inotropic support, extracorporeal membrane oxygenation or a left ventricular assist device should be taken into consideration, depending on the clinical context, comorbidities, prognosis of cardiac and non-cardiac complications alongside the cancer type/stage.

In cases of pericarditis, the guideline recommendations, that should be applied, include pericardiocentesis of large effusions and tamponade.

Patients with suspected acute coronary syndrome, who are also receiving ICIs therapy, should be admitted to a coronary unit for continuous EKG monitoring, surveillance of cardiac biomarkers and of the left ventricular function including measurement of the left ventricular ejection fraction and strain. Beta-blockers and angiotensin-converting-enzyme-inhibitors have not been directly correlated to the inhibition of the emergence of adverse cardiac events related to ICIs. However, all this medication should be administered in cases of left ventricular dysfunction. Moreover, in compliance with the recommendations made in the current ESC guidelines on the management of acute coronary syndromes, a coronary angiography should be performed when an acute coronary syndrome is suspected (28).

### Immunosuppression

The intensity of immunosuppression therapy depends on the severity of the adverse cardiac event, as described above. High intensity corticotherapy should be considered for severe cases of myocarditis, symptomatic heart failure, complete A-V block, ventricular arrhythmias (e.g., 500–1,000 mg/day i.v. methylprednisolone until the patient is clinically stable, followed by 1 mg/kg/day oral prednisolone with weaning, depending on the clinical course of the complication and periodical evaluation of troponin, inflammation on MRI, left ventricular dysfunction on echocardiogram, EKG).

If cortisteroid therapy is not sufficient, second line treatment with infliximab or mofetil should be considered. Immunoglobulin or anti-thymocyte globulin might be considered in extreme cases too (11, 28).

## Conclusion

Having all these considered, it becomes clear that before initiating ICIs treatment in a cancer patient, a baseline accurate cardiac examination is required. This examination should include clinical workup combined with a serum biomarker report, an ECG and an echocardiogram that will provide information on the LV ejection fraction and strain measurements as well. The appropriate time between tests remains unclear, but currently we have an ongoing project, in which we are testing a set of biomarkers in conjunction with some echo parameters in order to be able to assess cardiac toxicity related to ICIs before it is too late for the patient's well-being. As literature confirms, cardiac troponin and NT-proBNP can be chronically elevated in some subsets of patients. Therefore, we have chosen other types of biomarkers in order to be able to detect LV dysfunction before the onset of decreased EF. Much is still unknown about the ICIs-related cardiotoxicity and therefore, further research is required.

## Author Contributions

DM-H, AF, RM-H, AP, and AB contributed equally to this work. DM-H, AF, and ST research the literature. RM-H, AP, AF, and AB studied and analysed the articles. DM-H, AF, RM-H, ST, AP, and AB wrote the paper. All authors contributed to the article and approved the submitted version.

## Funding

This research was funded by Knowledge transfer of bio-genomics in oncology and related domains in clinical applications – BIOGENONCO, MySMIS Code: 105774, Financing contract No: 10/01.09.2016.

## Conflict of Interest

The authors declare that the research was conducted in the absence of any commercial or financial relationships that could be construed as a potential conflict of interest.

## Publisher's Note

All claims expressed in this article are solely those of the authors and do not necessarily represent those of their affiliated organizations, or those of the publisher, the editors and the reviewers. Any product that may be evaluated in this article, or claim that may be made by its manufacturer, is not guaranteed or endorsed by the publisher.
